# Supplementary material for: Iterative improvement in the automatic modular design of robot swarms
Source: PeerJ Comput Sci. 2020 Dec 7;6:e322. doi: 10.7717/peerj-cs.322 (PMC7924708; doi:10.7717/peerj-cs.322)
Supplement: Supplemental Information 3 [file peerj-cs-06-322-s003.zip › argos3/doc/api/standalone/a00497_source.html]

ARGoS: plugins/robots/generic/control\_interface/ci\_gripper\_actuator.h Source File


- Main Page
- Related Pages
- Namespaces
- Classes
- Files

- File List
- File Members

# plugins/robots/generic/control\_interface/ci\_gripper\_actuator.h

Go to the documentation of this file.

```
00001 
00007 #ifndef CCI_GRIPPER_ACTUATOR_H
00008 #define CCI_GRIPPER_ACTUATOR_H
00009 
00010 namespace argos {
00011    class CCI_GripperActuator;
00012 }
00013 
00014 #include <argos3/core/control_interface/ci_actuator.h>
00015 
00016 namespace argos {
00017 
00018    class CCI_GripperActuator : public CCI_Actuator {
00019 
00020    public:
00021 
00025       CCI_GripperActuator();
00026 
00030       virtual ~CCI_GripperActuator() {}
00031 
00038       void SetLockState(Real f_lock_state);
00039 
00045       void Lock();
00046 
00052       void Unlock();
00053 
00054 #ifdef ARGOS_WITH_LUA
00055       virtual void CreateLuaState(lua_State* pt_lua_state);
00056 #endif
00057 
00058    protected:
00059 
00060       Real m_fLockState;
00061 
00062    };
00063 
00064 }
00065 
00066 #endif
```

---

Generated on 10 Jul 2018 for ARGoS by 
 1.6.1
